# Changes in extracellular water with hemodialysis and fall in systolic blood pressure

**DOI:** 10.1177/0391398821995503

**Published:** 2021-02-20

**Authors:** Kamonwan Tangvoraphonkchai, Andrew Davenport

**Affiliations:** 1 Department of Renal Medicine, Mahasarakham University Hospital, Mahasarakham, Thailand; 2 UCL Department of Nephrology, Royal Free Hospital, University College London, London, UK

**Keywords:** Dialysate sodium, hemodialysis, extracellular water, intradialytic hypotension

## Abstract

**Introduction:**

Intra-dialytic hypotension (IDH) remains the most common complication with outpatient hemodialysis (HD) sessions. As fluid is removed during HD, there is contraction of the extracellular volume (ECW). We wished to determine whether the fall in ECW was associated with a fall in systolic blood pressure (SBP).

**Methods:**

We retrospectively reviewed the records of adult dialysis outpatients attending for their midweek sessions who had corresponding pre- and post-HD bioimpedance measurements of ECW.

**Result:**

We reviewed 736 patients, median age 67 (54–76) years, 62.8% male, 45.7% diabetic with a median dialysis vintage of 24.4 (9.2–56.8) months. The percentage fall in ECW (ECW%) was associated with post-dialysis systolic blood pressure (SBP) (*r* = −0.14, *p* < 0.001). Patients with SBP falls of >20 mmHg had a greater fall in ECW% compared to patients with stable SBP 7.6 (4.6–10.1) vs 6.0 (4.0–8.5), *p* < 0.001). Patients with greater dialyzer urea clearance had greater fall in ECW% (*r* = 0.19, *p* < 0.001). In a logistic model an increased fall in ECW% was associated with weight loss (odds ratio (OR) 1.88, 95% confidence limits (CL) 1.62–2.176, *p* < 0.001), and session duration (OR 1.45 (CL 1.05–1.99), *p* = 0.024), and negatively with hemodiafiltration compared to hemodialysis (OR 0.37 (0.19–0.74) *p* = 0.005 and dialysate sodium to plasma gradient (OR 0.95 (CL 0.90–0.99), *p* = 0.021).

**Conclusion:**

We observed an association between the reduction in ECW and SBP with dialysis. Our results would advocate monitoring ECW changes during dialysis and developing biofeedback devices to control ultrafiltration and dialysate sodium to reduce the risk of IDH.

## Introduction

Intradialytic hypotension (IDH) is the most commonly reported complication of routine outpatient hemodialysis (HD) treatments, with a reported incidence of 10–69%, depending on definition,^1^ Several studies have reported IDH to be a risk factor for mortality.^2–5^ The pathophysiology of IDH is multi-factorial, but the simplest explanation would be that ultrafiltration leads to a reduction in the effective circulating volume, with the rate of fluid removal exceeding the rate at which fluid can be mobilized to refill the circulating volume.. Thus, maintaining a stable circulating volume during HD might attenuate or reduce the incidence of IDH and potentially prevent adverse outcomes. Preliminary studies reported that by monitoring changes in hematocrit could reduce IDH, and other intra-dialytic symptoms, and potentially reduce patient mortality.^6^ However, a multi-center prospective study, the Crit-Line Intradialytic Monitoring Benefit (CLIMB) Study, not only failed to demonstrate a beneficial advantage for monitoring changes in plasma volume, but also reported more complications with hospital admissions.^7^ However, later smaller studies of patients prone to IDH, reported that reacting to changes in the circulating volume, by monitoring the hematocrit coupled with compensatory changes in ultrafiltration and dialysate sodium, reported a reduction in the severity of IDH.^8^ As such, we wished to review changes in extracellular water (ECW), and whether there was an association with changes in peri-dialytic blood pressure.

## Material and methods

We reviewed the dialysis records of 736 adult patients attending for routine outpatient mid-week dialysis sessions, who had corresponding bioimpedance measurements pre-and post-dialysis, and had no nursing interventions during the session. .All patients received standard dietary advice to restrict dietary sodium intake to around 100 mmol/day. Patient co-morbidity was assessed uding the United Kingom (UK) Stoke-Davies co-morbidity grading.^9^ Center practice was to prescribe furosemide to all patients with a urine output >200 mL/day.

Patients dialyzed with Fresenious 4008 H and BBraun Dialogue R+ machines (Fresenius Bad Homberg and BBraun, Melsungen, Germay), using polysulfone dialyzers, median dialyzer size 1.8 m^2^ (1.8–2.2 m^2^), ultrapure water quality and anticoagulated with bolus tinzaparin (Leo Laboratories, Risborough, UK).^10,11^ All patients were dialyzed with a constant ultrafiltration profile. Dialysate sodium calibrated with a conductance meter against standards.^12^ Patients were requested not to eat during treatment and were restricted to a maximum 150 mL drink during the dialysis session.

Multi-frequency bioimpedance (Biospace in body 720, Seoul, South Korea) was performed pre and post-dialysis. Post dialysis measurements were performed in a standardized protocol and measured 20 min after dialysis disconnection in order to allow fluid redistribution.^13–15^ Bioimpedance measurements were not made in patients with pacemakers and other implantable cardac devices, and those with limb amputations, or paralysis.

Serum biochemistry samples were analyzed with a standard multi-channel biochemical analyzer (Roche Integra, Roche Diagnostics, Lewes, UK), using an indirect ion selective electrode for sodium, the bromocresol green method for albumin determination, and hemoglobin using a standard analyzer (XE-2100 Sysmex Corporation, Kobe, Japan).^16^ The dialysate to serum sodium gradient was calculated as the difference between the prescribed dialysate sodium and pre-dialysis serum sample. The percentage change in ECW reduction was calculated by the following formula:

$$\begin{array}{l} \%\mathrm{ECW}\;\mathrm{reduction}=\;(\mathrm{Pre}-\mathrm{dialysis}\;\mathrm{ECW}\\ \;\;\;\;-\mathrm{Post}-\mathrm{dialysis}\;\mathrm{ECW})\;*100/\mathrm{Pre}-\mathrm{dialysis}\;\mathrm{ECW} \end{array}$$

## Statistical analysis

Results are expressed as mean ± standard deviation, or median and interquartile range, or percentage. Variables were tested for normality by the Kolmogorov-Smirnov and analyzed using Kruskal Wallis with appropriate post-hoc testing. Spearman correlation used to assess univariate association and we constructed logistic regression models based on the median change in ECW to determine associates of %ECW reduction, using a enter method including variables those thought to be clinically relevant. Analyses were performed with SPSS 23 (SPSS 23, IBM, Armonk, New York, USA). Statistical significance was taken as *p* < 0.05.

## Ethics

Our retrospective audit complied with the UK National Health Service Health Research Authority guidelines for clinical audit and service development with all patient data anonymized prior to analysis (https://www.hra.nhs.uk), and was registered with the University College London department of nephrology.

## Results

We reviewed the mid-week dialysis records of 736 HD outpatients who had corresponding pre- and post-dialysis bioimpedance measurements and had not suffered symptomatic IDH requiring an intervention with intra-venous fluids, or a reduction in ultrafiltration rate, median age 67 (54–76), 62.8% male, 45.7% diabetic and a median dialysis vintage of 24.4 (9.2–56.8) months. Most patients had a history of hypertension (86.1%), 34.2% pre-existing heart disease, 14.5% stroke and 36% of patients were prescribed diuretics. The median Davies comorbidity score was 1.5 (1–2). The majority of patients (92.9%) were treated by on-line-hemodiafiltration (OL-HDF).

We divided patients into three groups based on the reduction in ECW following dialysis (Table 1). Those patients with the greatest reduction in ECW were younger, of longer dialysis vintage, with longer dialysis session times, higher pre-dialysis serum urea, creatinine and potassium, greater weight reduction, with greater ultrafiltration requirements and fewer patients were prescribed diuretics and treated by OL-HDF .

**Table 1.** Characteristics of patients according to the reduction in extracellular water (ECW) with the first tertile having the smallest fall and the third tertile having the largest fall in ECW.

Change in ECW	Smallest change	Middle tertile	Greatest change	*p*
Number of patients	245	246	245	
Age, years	68 (58–78)	68 (54–76)	64 (51–75)	0.017
Male (%)	154 (62.9)	154 (62.6)	154 (62.9)	0.998
Weight pre, kg	68.8 (60.8–82.5)	73.1 (62.6–84.3)	70.1 (59.8–80.2)	0.125
Dialysis vintage, month	21 (7–54)	23 (10–54)	27 (12–62)	0.025
Hypertension (%)	212 (86.5)	218 (88.6)	204 (83.3)	0.224
Heart disease (%)	85 (34.7)	88 (35.8)	79 (32.2)	0.7
Diabetes (%)	120 (49)	111 (45.1)	105 (42.9)	0.338
Davies comorbidity score	2 (1–2)	2 (1–2)	1 (1–2)	0.398
Diuretics (%)	98 (40)	96 (39)	71 (29)	0.019
Session time, hours	3.75 (3–4)	3.75 (3.5–4)	4 (3.5–4)	<0.001
On-line HDF (%)	235 (95.9)	232 (94.3)	217 (88.6)	0.004
SBP post dialysis, mmHg	134 (115–151)	130 (115–154)	122 (107–139.5)	<0.001
DBP post dialysis, mmHg	69 (58–78.5)	69 (60–80)	68.5 (57–80)	0.314
SBP drop >20 mmHg %	69 (28.2)	76 (30.9)	109 (45)	<0.001
ECWpost, L	14.2 (11.9–17.3)	14 (12.1–16.2)	12.8 (10.8–15.2)	<0.001
ECW/TBWpost dialysis, ratio	0.397 (0.388–0.409)	0.395 (0.384–0.404)	0.389 (0.378–0.403)	<0.001
Percent weight change, %	1.9 (1.1–2.6)	2.7 (2–3.4)	3.1 (2.5–3.8)	<0.001
Ultrafiltration rate ml/min	6.1 (4.0–8.2)	9.0 (6.7–11.1)	10.0 (7.6–11.7)	<0.001
Ultrafiltration rate ml/min//kg	0.086 (0.052–0.123)	0.123 (0.088–0.156)	0.141 (0.108–0.166)	<0.001
Serum sodium, mEq/L	139 (137–141)	139 (137–141)	139 (136–141)	0.633
Serum potassium, mEq/L	4.9 (4.5–5.4)	5 (4.6–5.5)	5.2 (4.8–5.7)	<0.001
Serum urea, mmol/L	17.5 (14.1–20.9)	18.5 (14.8–21.9)	19 (14.6–22.7)	0.02
Serum creatinine, mmol/L	628 (519–811)	692.5 (554–856)	718.5 (573–868)	0.005
Serum bicarbonate, mEq/L	22 (20–24)	22 (20–23)	22 (20–23)	0.429
Haemoglobin, g/dL	10.9 (9.8–11.9)	10.9 (10.1–11.8)	11.2 (10.2–12)	0.304
Serum Albumin, g/L	38 (36–41)	40 (37–42)	40 (37–42)	0.003
Serum NT-proBNP, pg/ml	454 (187–1366)	458 (156–1627)	432 (153–1203)	0.663
C-reactive protein, mg/L	6 (2–16)	5 (2–10)	5 (2–12)	0.03
B2-microglobulin, µg/mL	27.2 (21.2–33)	28.4 (22.8–33.8)	28.9 (23.3–33.7)	0.168
Urea reduction, %	74.6 (67.8–78.6)	74.3 (69.5–78)	76.1 (71.9–80)	0.001
Dialyser Kt/Vurea	1.57 (1.28–1.77)	1.57 (1.37–1.79)	1.7 (1.5–1.9)	<0.001
nPNA, g/kg/day	1.57 (1.28–1.77)	0.98 (0.8–1.14)	1.02 (0.83–1.2)	<0.001
Dialysate sodium, mEq/L	137 (136–138)	137 (136–138)	137 (136–138)	0.008
D-S sodium gradient	−1 (−4 to −1)	−2 (−4 to −0)	−2 (−4 to −1)	0.358

HDF: Hemodiafiltration; SBP and DBP: systolic and diastolic blood pressure; TBW: total body water; NT-proBNP: N terminal pro-natriuretic peptide; nPNA: normalized protein nitrogen appearance; (D-S) Na: dialysate to serum sodium gradient.

Results as integer, percentage, mean ± standard deviation or median (interquartile range). *p* value versus first tertile.

On univariate analysis the % reduction in ECW was associated with nutrional factors; including normalized protein nitrogen appearance (nPNA), and pre-dialysis serum urea, creatinine, albumin (Table 2), and younger patients and those with a longer dialysis vintage, and dialysis session factors, including weight loss, ultrafiltration rates, sessional time, and both urea reduction and dialyzer urea clearance (Kt/Vurea). Although there was a significant correlation between the change in ECW and ultrafiltration rate, less than15% in the variance of ECW could be explained by the ultrafiltration rate. In addition the change in ECW was smaller with OL-HDF compared to HD, and for those patients prescribed diuretics. A greater percentage ECW reduction resulted in a greater fall in ECW/TBW ratio, lower post-dialysis systolic blood pressure (SBP), and more patients with a greater fall in SBP of 20 mmHg or greater. Whereas a history of cardiac disease, serum N-terminal brain natriuretic peptide (NT-proBNP),and diabetes were not significantly associated with a change in ECW (all *p* > 0.05). Although there was a statistical difference in dialysate sodium between the tertiles of change in ECW, the median and interquartile ranges were identical. On univariate analysis there was no statistcial association betweent he change in ECW and serum sodium, dialysate sodium or the dialysate to serum sodium gradient.

**Table 2.** Univariate association between percentage ECW reduction and patient’s factors.

Variables	% Reduction in ECW	
	Rho or median of %ECW reduction	*p*
Ultrafiltration rate, ml/min	0.384	<0.001
Ultrafiltrate rate ml/min//kg	0.381	<0.001
Dialyzer Kt/Vurea	0.194	<0.001
nPNA, g/kg/day	0.163	<0.001
Session time, hours	0.156	<0.001
SBP post, mmHg	−0.144	<0.001
SBP drop >20 vs none	7.6 (4.6–10.1) vs 6.0 (4.0–8.5)	<0.001
Percent weight change, %	0.143	<0.001
On-line HDF vs HD	6.4 (4–9) vs 8.3 (5–13)	0.001
Urea reduction ratio, %	0.127	0.001
Dialysis vintage, month	0.11	0.003
Serum creatinine, mmol/L	0.107	0.004
Age, years	−0.101	0.006
Diuretic vs none	6.1 (3.8–8.4) vs 6.8 (4.3–9.5)	0.007
Serum albumin, g/L	0.097	0.009
Serum urea, mmol/L	0.092	0.012

HDF: Hemodiafiltration; SBP and DBP: systolic and diastolic blood pressure; TBW: total body water; NT-proBNP: N terminal pro-natriuretic peptide; nPNA: normalized protein nitrogen appearance.

As a minority of patients had an increase in ECW, as not all patients had weight loss with the dialysis session, we generated a logistic regression model comparing variables above and below median percentage change in ECW (Table 3). A greater percentage fall in ECW was independtly associated with percentage weight loss and longer dialysis session time, and negatively with the use of OL-HDF compared to conventional HD and the dialysate to serum sodium gradient (D-S Na)

**Table 3.** Logistic regression model of factors associated with % ECW reduction beta (β), standard error β (SE β), odds ratio (OR), 95% confidence limits (95%CL) Nagelkerke *r*^2^ = 0.213.

Variables	β	SE of β	OR of more ECW volume reduction	95%CL	*p* Value
D-P Na gradient	−0.057	0.025	0.945	0.901–0.992	0.021
Weight loss (%)	0.631	0.075	1.879	1.622–2.176	<0.001
Age years	−0.007	0.005	0.993	0.982–1.003	0.167
Dialysis session time	0.371	0.164	1.450	1.051–1.999	0.024
On-line HDF	−0.983	0.346	0.374	0.190–0.738	0.005
Pre-dialysis serum urea	0.026	0.014	1.026	0.998–1.056	0.071
Vintage months	0.001	0.001	1.001	0.998–1.004	0.411
Diuretic usage	−0.276	0.173	0.758	0.54–1.066	0.111

## Discussion

Intradialytic hypotension (IDH) is a the most common complication of outpatient HD treatments.^17^ The incidence of symptomatic IDH has been estimated to occur in 20–30% of hemodialysis sessions.^18^ We reviewed bioimpedance measurements of ECW pre- and post-dialysis in more than 700 dialysis outpatients attending for their mid-week dialysis session. We excluded patients with symptomatic hypotension, as administration of fluids, or change in ultrafiltration rate could have altered the post sessional measurement of ECW. Those patients with the greatest reduction in ECW post-dialysis had greater falls in SBP, and more of these patients had a fall in SBP of ⩾20 mmHg. Not surprisingly there was a positive correlation between weight loss and the percentage reduction in ECW. However, on one hand younger patients and those with greater pre-dialysis serum urea, creatinine, potassium and albumin with a higher nPNA had a greater fall in ECW on univariate analysis, suggesting that younger patients, and those with greater dietary protein intake may have had greater weight gains between dialysis sessions, so requiring more fluid and urea removal and longer dialysis sessions. Although a higher ultrafiltration rate was associated with an increased change in ECW, the variance was less than 15%, so many other factors contribute to the change in ECW, and ultrafiltration rate was removed from the step-backward logistical model as it was not statistically significant and did not improve the model fit.

We also noted that patients treated by OL-HDF had a smaller change in ECW compared to those treated by HD. Although previous smaller studies did not observe a difference in ECW between dialysis modes,^19,20^ there has been a change in clinical practice with centers now aiming for larger convection volume exchange targets.^21^ There have been reports that sodium balance may differ between hemofiltration and HD, and so smaller changes in ECW may be due to less sodium removal.^22^ Although differences in thermal balance between HD and OL-HDF may equally affect changes in ECW. In keeping with other reports patients with residual renal function prescribed diuretics had smaller changes in ECW,^23^ and supporting the contention that one of the benefits of maintaining residual renal function is to prevent volume overload.^24^

Several different interventions or combinations have been introduced to reduce the risk of IDH, ranging from cooling the dialysate, ultrafiltration profiling, increased dialysate sodium concentrations, administration of alpha-adrenergic agonists, or more frequent and longer hemodialysis sessions.^18^

Selecting a higher dialysate sodium concentration compared to plasma sodium has been reported to improve hemodynamic stability and prevent intradialytic symptoms.^25^ Although our study would support this,the prolonged use of higher dialysate sodium concentrations could lead to greater weight gains between dialysis sessions.^26^ However, balance studies have suggested that to have a detectable effect, the dialysate sodium needs to be ⩾5 mmEq/L greater than the serum sodium,^27^ and studies using a biofeedback adjusting dialystae sodium according to changes in relative blood volume (RBV) reduced the incidence of symptomatic IDH, but wiithout evidence of excess inter-dialytic weigh gains.^8^

While we found no association between NTproBNP and the change in ECW or SBP, other studies also failed to demonstrate a relationship between cardiac chamber sizes and ECW and SBP.^28,29^ However our study does reinforce the relationship between a change in ECW and reduction in SBP during dialysis. Although a multicenter trial of monitoring RBV failed to demonstrate a benefit,^7^ other studies have shown that coupling RBV with biofeedback systems designed to adjust ultrafiltration and dialysate sodium have reported a reduction in the frequency and severity of IDH and reported that biofeedback driven interventions can reduce the incidence of IDH.^8,30^

As with any observational cross-sectional study, we can only report associations and formulate a hypothesis, but not determine causality. We only studied patients with pre and post-dialysis bioimedance measurements and as such patients with intracardiac pacing and other devices were excluded, so patients with the most severe cardiac dysfunction were not studied. Similarly, we excluded patients with symptomatic hypotension which required a nursing intervention, as this would have altered the change in ECW. However this makes our findings more applcable to the general patient attending for dialysis. As only a minority of patients were treated by HD, further studies would be required to confrm that hemodiafiltration leads to smaller changes in ECW than hemodialysis.

Our study is the largest report of changes in ECW during a mid-week outpatient dialysis session, and we have demonstrated that changes in ECW are associated with corresponding changes in SBP, when using a constant ultrafiltration profile. In addition to symptomatic IDH, it is now recognized that asymptomatic falls in SBP are associated with increased morbidity and mortality. We found that the reduction in ECW was associated with weight loss, and an increasing dialysate to serum sodium gradient, and less with OL-HDF. Thus, our report confirms an association between a reduction in ECW and SBP, and by demonstrating the effect of weight loss by ultrafiltration and dialysate sodium gradient supports the further development of biofeedback devices designed to monitor changes in ECW, and then adjust ultrafiltration rates and dialysate sodium concentrations.
